# Lung and Colorectal Cancer Disparities in Appalachian Kentucky: Spatial Analysis on the Influence of Education and Literacy

**DOI:** 10.3390/ijerph20146363

**Published:** 2023-07-14

**Authors:** Nicole M. Robertson, Todd Burus, Lauren Hudson, Pamela C. Hull, Lee Park, Nathan L. Vanderford

**Affiliations:** 1 College of Medicine, University of Kentucky, Lexington, KY 40536, USA; nicole.robertson@uky.edu (N.M.R.); lauren.hudson@uky.edu (L.H.); pam.hull@uky.edu (P.C.H.); 2 Markey Cancer Center, University of Kentucky, Lexington, KY 40536, USA; tburus@uky.edu; 3 Department of Behavioral Science, College of Medicine, University of Kentucky, Lexington, KY 40536, USA; 4 Department of Statistics, College of Arts and Sciences, University of Kentucky, Lexington, KY 40536, USA; lee.park@uky.edu; 5 Department of Toxicology and Cancer Biology, College of Medicine, University of Kentucky, Lexington, KY 40536, USA

**Keywords:** Appalachia, cancer, rural health, cancer education, literacy, disparities

## Abstract

Low educational attainment and high cancer incidence and mortality rates have long been a challenge in Appalachian Kentucky. Prior studies have reported disparities in cancer incidence and mortality between Appalachian and non-Appalachian populations, but the influence of education on this disparity has not been extensively studied. Herein, all cancers and two cancer sites with available screenings (colorectal and lung) were joined with education indicators (educational attainment and literacy) and one geographic indicator across all 120 Kentucky counties. This dataset was used to build choropleth maps and perform simple linear and spatial regression to assess statistical significance and to measure the strength of the linear relationship between county-level education and cancer-related outcomes in Appalachian and non-Appalachian Kentucky. Among all cancer sites, age-adjusted cancer incidence and mortality was higher in Appalachian versus non-Appalachian Kentucky. The percentage of the population not completing high school was positively correlated with increased colorectal and lung cancer incidence and mortality in Appalachia. Similarly, counties with a higher percentage of the population lacking basic literacy had the strongest correlation with colorectal and lung cancer incidence and mortality, which were concentrated in Appalachian Kentucky. Our findings suggest a need for implementing interventions that increase educational attainment and enhance basic literacy as a means of improving cancer outcomes in Appalachia.

## 1. Introduction

The Appalachian region has a population of 25 million and is characterized as a rural mountainous region of the Eastern United States spanning 13 states as far north as New York and as far south as Mississippi. The Appalachian region is historically marked by high poverty, high unemployment rates, low educational attainment, low health literacy, and poor health outcomes [1,2,3]. Specifically, the Appalachian region has markedly higher cancer incidence and mortality rates compared to non-Appalachian counties [4,5,6]. In Kentucky, the age-adjusted cancer incidence rate is the highest in the nation for all cancer sites at 513.8 cancer cases per 100,000 people compared to the national rate of 438.3 cases per 100,000 people among all cancer sites [7]. The prior literature has shown that cancer incidence was significantly higher in Appalachian populations compared to non-Appalachian populations [7,8]. Relative to urban non-Appalachian residents, the age-adjusted cancer mortality rate is reported up to 15–36% higher in Appalachian residents in Kentucky, West Virginia, Alabama, Tennessee, Mississippi, Ohio, and Virginia [6].

In Kentucky, disparities in cancer incidence and mortality between Appalachian and non-Appalachian populations are most prominent in colorectal and lung cancers, which have affordable screening modalities, have improved survival if diagnosed at earlier stages, and—in the case of colorectal cancer screening (e.g., colonoscopy)—can prevent cancer through removing pre-cancerous polyps. Lung cancer currently leads all cancer sites in the highest estimated number of new cases and deaths among both sexes in 2022 in Kentucky [9]. The 2021 Kentucky Cancer Needs Assessment [7] reported that age-adjusted cancer incidence was also significantly higher in the Appalachian Kentucky population relative to Kentucky for colorectal and lung cancer with the increase in cancer incidence being 12.2% and 18.4%, respectively [7]. Similarly, in Kentucky, the age-adjusted mortality rate for lung cancer was substantially higher in Appalachian populations relative to the remainder of the state, with a percent difference of 18.2% [7]. Likewise, the age-adjusted mortality rate for colorectal cancer was significantly higher in Appalachian populations relative to the non-Appalachian population with an increase of 14.1% [7].

These colorectal and lung cancer disparities in Appalachian populations have been attributed to a complex interplay of increased prevalence of obesity, tobacco use, alcohol and substance abuse, environmental exposure, lack of access to healthcare services, and low educational attainment [8,10,11]. More specifically, lower educational attainment has been shown to be strongly associated with increased mortality rates in the U.S. [12]. However, educational attainment as a risk factor for increased colorectal and lung cancer incidence and mortality has not been extensively studied in Appalachian Kentucky. In the U.S., Kentucky ranked 45th in bachelor’s degree attainment in 2020 [13]. Currently, the percentage of the population with a high school education is 79.0% among Appalachian residents in Kentucky compared to the national average at 88%. Appalachian Kentucky has the lowest post-secondary education attainment of the Appalachian region at 22.8% [14]. Further disparities exist in Central Appalachia, consisting mainly of Eastern Kentucky, where 15.2% of residents have attained a bachelor’s degree compared to the national average of 33.5% [15]. Since 2010, there has been minimal rise in both high school diploma and bachelor’s degree attainment in the Appalachian region, continuing to lag substantially behind the U.S. average [15,16]. It is difficult to focus on college preparedness for all high school students in the Appalachian region of Kentucky where the limited number of available jobs do not require a college education. The landscape of high unemployment rates and lack of access to jobs further influences Appalachian students as they transition from high school to college and consider career options requiring a college degree [17]. This may further perpetuate a cycle of lower educational attainment in rural Appalachian communities relative to urban non-Appalachian counterparts and further lead to disparities in cancer outcomes.

Low educational attainment rates in the Appalachian region further contribute to low basic literacy, and more specifically health and cancer literacy [14]. Additionally, educational attainment has been used as a surrogate measure for basic literacy, which is a known marker for increased cancer screening knowledge and participation [18,19,20]. Additional studies have shown that lower literacy was associated with poorer quality of life and cancer care [12,21,22]. However, no studies to date have investigated the influence of educational attainment and basic literacy on cancer incidence and mortality in Appalachia. This demonstrates the need for further investigation into the influence of educational attainment and literacy on cancer outcomes in at-risk populations such as in Appalachia [10]. With a high incidence and mortality of lung and colorectal cancer in the U.S., which is higher in Appalachia, addressing socioeconomic factors related to education and literacy may be a strategy to reduce the burden of these cancers in this population [23].

The objective of this study is to evaluate the relationship between colorectal and lung cancer incidence and mortality outcomes and educational attainment and literacy between Appalachian and non-Appalachian counties in Kentucky at the county level. Understanding the geographic distribution of educational attainment and literacy as it relates to cancer incidence and mortality throughout Kentucky can inform colorectal and lung cancer prevention efforts and efforts to target increased educational attainment and literacy in Appalachian communities as a means of reducing cancer disparities.

## 2. Materials and Methods

A dataset was created that contained information for each of Kentucky’s 120 counties on age-adjusted colorectal and lung and bronchus cancer incidence and mortality; various indicators of population educational attainment and literacy; and Appalachian designation. Appalachian designation was assigned in accordance with 2021 listings from the Appalachian Regional Commission. Among the 54 Appalachian counties in Kentucky, 93% of these counties are designated non-metro according to the Economic Research Service 2013 Rural-Urban Continuum Codes, which is a classification scheme in distinguishing rurality of U.S. counties [24]. As such, our study setting focuses on a majority non-metro, rural population in Kentucky. We chose to evaluate colorectal and lung and bronchus cancer for this study as screenable cancers supported by U.S. Preventive Services Task Force (USPSTF) guidelines. Additionally, both colorectal and lung cancer were selected due to the burden of these cancers in Appalachian communities and increased screening uptake of these cancers following community-based outreach and educational interventions in Appalachia [25]. Prior literature has also found increasing colorectal and lung cancer mortality rates with decreasing years of education among non-Hispanic White participants, but this trend was not noted in breast cancer [12].

### 2.1. Cancer Data

Cancer incidence and mortality data were obtained from the Kentucky Cancer Registry (KCR), a population-based central cancer registry for the Commonwealth of Kentucky. Given that all healthcare facilities in the state that diagnose or treat cancer are mandated by the Commonwealth of Kentucky to report all new cancer diagnoses to KCR, KCR data represent the entire population of new cancer cases in the state. As a member of the National Cancer Institute’s Surveillance, Epidemiology, and End Results (SEER) program since 2000, KCR meets the highest standards of accuracy and completeness for cancer registries. Rates for the years 2014–2018 were aggregated and age-adjusted according to the 2000 U.S. Standard Million Population. Complete data were available for all variables on all counties except for colorectal cancer mortality, which was suppressed on two counties due to county death totals being less than 5 in the specified time frame. Colon and rectum cancer will be referred to as colorectal cancer and lung and bronchus cancer will be referred to as lung cancer throughout the paper.

### 2.2. Education Data

County level population percentages of those age 25 or older who did not attend high school, who did not complete high school, and who did not complete college were gathered from the American Community Survey 5-Year Estimates for 2014–2018 using version 0.11.4 of the “tidycensus” package in the R software environment. Basic literacy describes the English literacy of America’s adults 16 years and older specifically in literacy, numeracy, and problem solving as measured by completion of the 2003 National Assessment of Adult Literacy (NAAL). This remains the most comprehensive measure of county-level adult basic literacy available nationally.

### 2.3. Visualizations

Individual variables from the dataset were visualized in county-level maps using quartiles. Pairs of variables were visualized in bivariate county-level maps using a 3 × 3 grid with tertiles. A boundary was placed around the Appalachian region for easy visual comparison. All geographic visualizations were built using ArcGIS Pro (version 2.9.1).

### 2.4. Statistical Analysis

Significance of cancer rate comparisons between U.S. versus Kentucky and non-Appalachian Kentucky versus Appalachian Kentucky were based on a rate ratio test [26]. Two county-level education variables (did not complete high school and lacking basic literacy in 2003) were considered as predictors and four county-level cancer variables (colorectal and lung cancer incidence and mortality) were used as outcomes to create eight different pairings for regression analysis on the 118 counties with complete data. Pairwise scatterplots were generated to assess linearity. Simple linear regression was run for each generating regression coefficients, the coefficient of determination, and appropriate *p*-values. For each model, residual plots and Q-Q plots were constructed to check regression normality assumption.

We assessed spatial autocorrelation in the linear models by calculating Moran’s I statistic on the residuals. Lagrange multiplier diagnostic tests were performed on models with a significant Moran’s I statistic to determine whether a spatial error or spatial lag model should be used to correct for spatial dependence, which we then fit to the data [27]. Spatial analysis was performed using the “spdep” package (version 1.2) in R.

## 3. Results

### 3.1. Colorectal and Lung Cancer Incidence and Mortality between Appalachian and Non-Appalachian Populations in Kentucky

Age-adjusted colorectal cancer incidence was substantially higher in the Appalachian Kentucky population at 54.8 cases per 100,000 people relative to the non-Appalachian population at 45.4 cases per 100,000 people in Kentucky, which was 20.7% higher in Appalachia (Table 1). Likewise, age-adjusted colorectal cancer mortality was 15.3 cases per 100,000 people in Appalachia compared to 13.5 cases per 100,000 people in non-Appalachian populations, which was 13.3% higher in Appalachia. We found the greatest disparity between Appalachian and non-Appalachian populations was in age-adjusted lung cancer incidence at 105.3 cases per 100,000 people and 82.4 cases per 100,000 people, respectively. Similarly, the age-adjusted lung cancer mortality was 48.4 deaths per 100,000 people in Appalachia relative to 37.5 deaths per 100,000 people in non-Appalachian populations. The increase between Appalachian and non-Appalachian populations in age-adjusted lung cancer incidence and mortality was 27.8% and 29.1%, respectively (Table 1).

### 3.2. Colorectal and Lung Cancer Incidence and Mortality by Geographic Distribution

**Figure 1:**
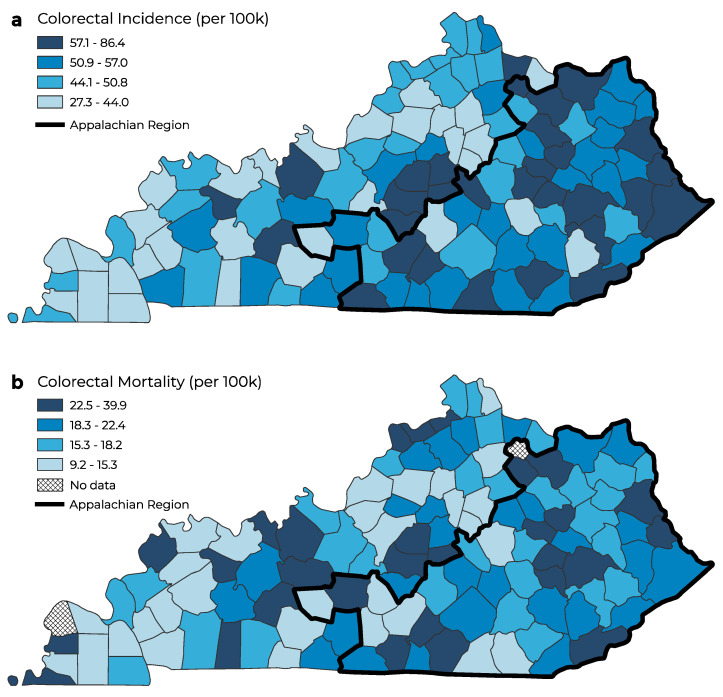
Geographic distribution of age–adjusted colorectal cancer incidence (**a**) and mortality (**b**) (per 100,000 people) in Kentucky.

**Figure 2:**
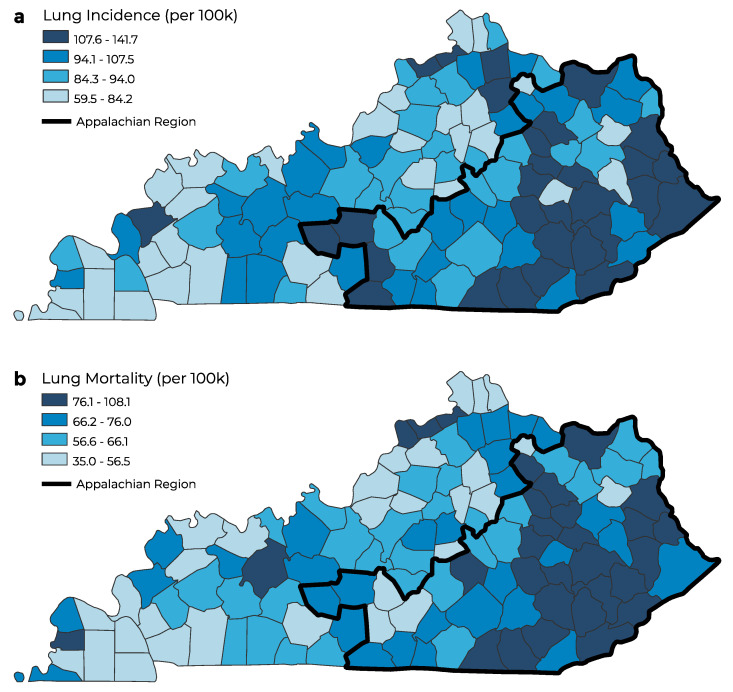
Geographic distribution of age–adjusted lung cancer incidence (**a**) and mortality (**b**) (per 100,000 people) in Kentucky.

The age-adjusted colorectal and lung cancer incidence rate varied by county in Kentucky (Figure 1a and Figure 2a). The highest age-adjusted cancer incidence was concentrated in eastern Kentucky, where the Appalachian counties in Kentucky are located. Similarly, the highest age-adjusted colorectal and lung cancer mortality rates were concentrated in the Appalachian region of Kentucky (Figure 1b and Figure 2b). Non-Appalachian counties had the lowest age-adjusted lung cancer incidence and mortality rates concentrated in southwestern, northern, and central Kentucky. The highest age-adjusted cancer incidence and mortality was concentrated in the Appalachian region of Kentucky for colorectal and lung cancers.

### 3.3. Cancer Rates and Educational Attainment in Appalachia Compared to Non-Appalachia Regions in Kentucky

**Figure 3:**
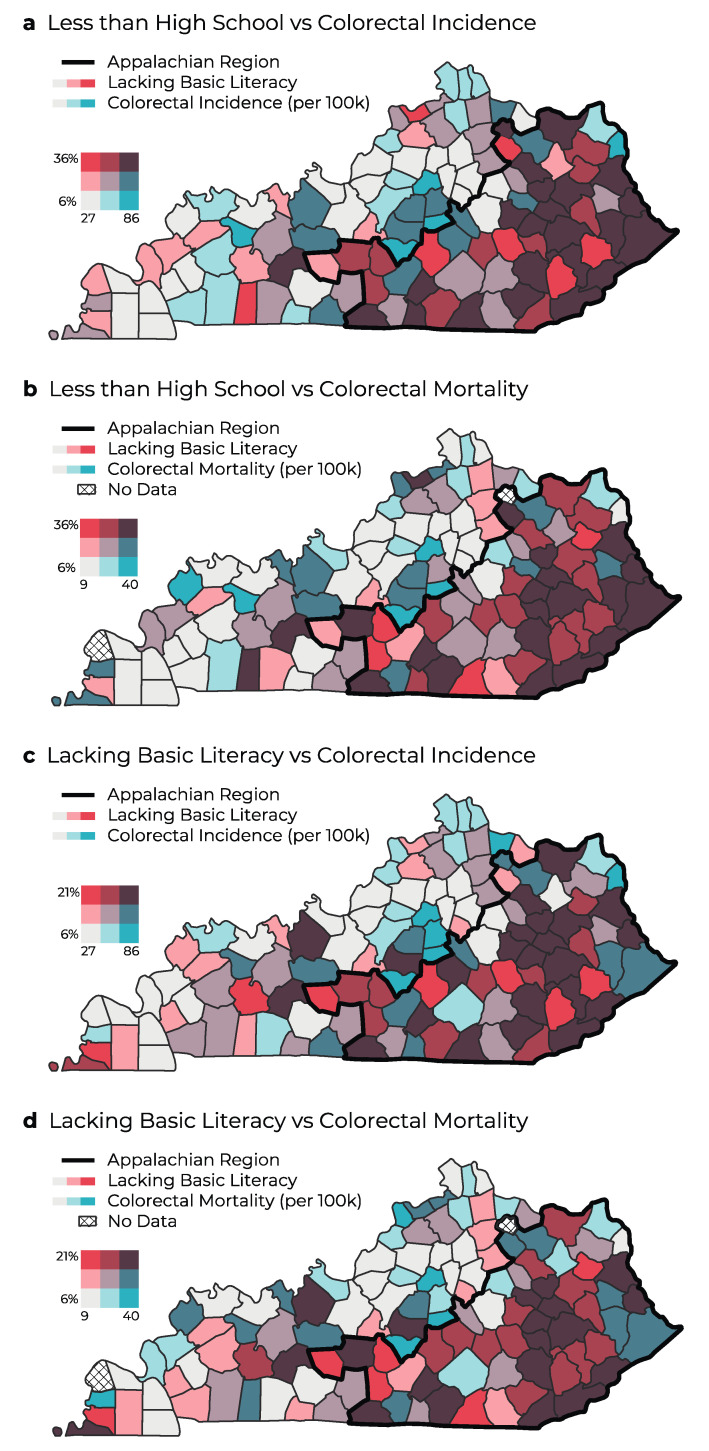
Geographic distribution and bivariate analysis of percentage of population with less than high school completion and colorectal cancer incidence (**a**) and mortality (**b**) and analysis of percentage of population lacking basic literacy (2003) and colorectal cancer incidence (**c**) and mortality (**d**) in Kentucky. Rates are per 100,000 people.

**Figure 4:**
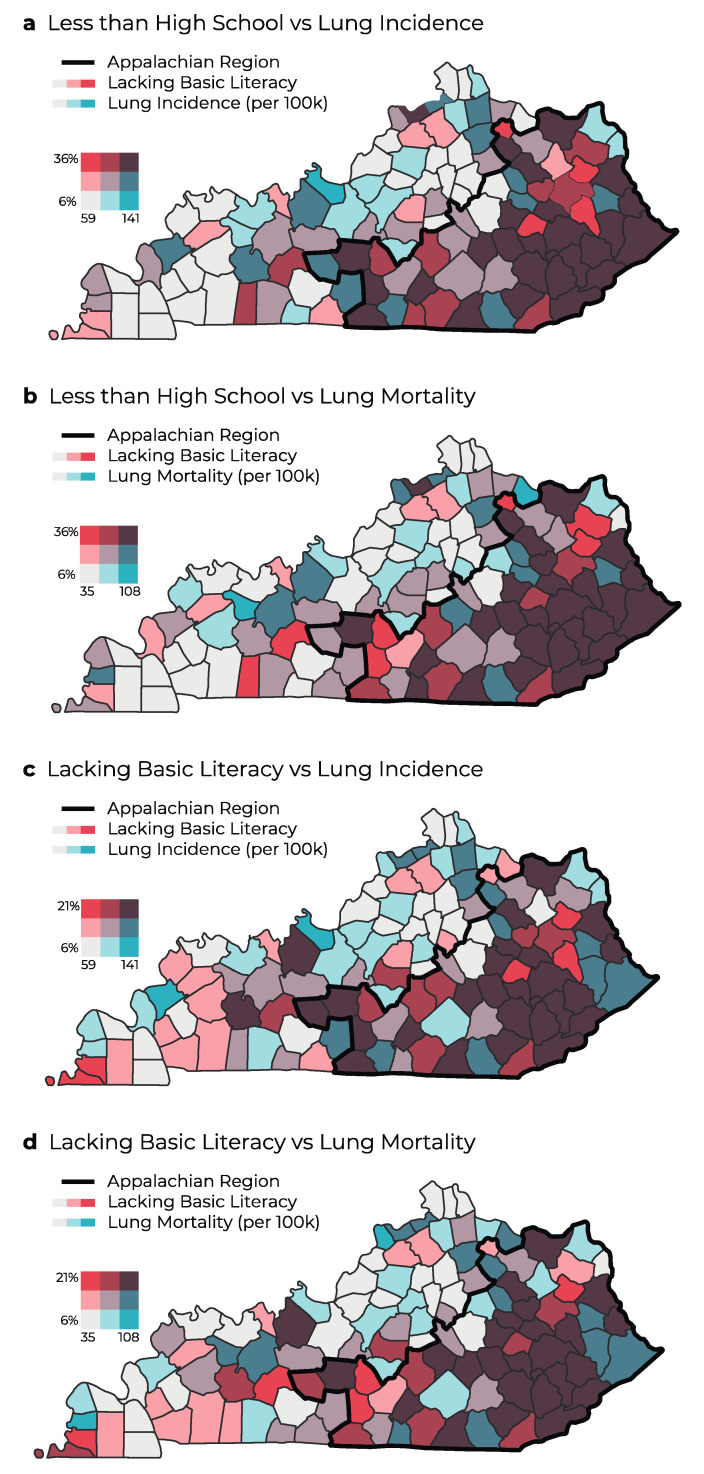
Geographic distribution and bivariate analysis of percentage of population with less than high school completion and lung cancer incidence (**a**) and mortality (**b**) and analysis of percentage of population lacking basic literacy (2003) and lung cancer incidence (**c**) and mortality (**d**) in Kentucky. Rates are per 100,000 people.

Through investigating associations between colorectal and lung cancer outcomes and educational attainment, we found that Appalachian counties had a strong correlation between cancer incidence and mortality and the percentage of less than high school completion. That is, as the percentage of the population with less than high school completion increased, the incidence and mortality of lung and colorectal cancers increased also (Figure 3a,b and Figure 4a,b). Counties with a large percentage of the population with less than high school completion had the highest colorectal and lung cancer incidence, which are concentrated in Appalachian Kentucky. Greater disparities were noted in lung cancer incidence and mortality between Appalachian and non-Appalachian counties (Figure 4a,b).

### 3.4. Cancer Rates and Basic Literacy in Appalachia Compared to Non-Appalachia Regions in Kentucky

In evaluating the correlation between cancer incidence and percentage of the population lacking basic literacy, we found the strongest correlation in Appalachian populations in Eastern Kentucky (Figure 3c,d and Figure 4c,d). This trend resembled a similar trend in colorectal and lung cancer mortality, which may reveal a positive relationship between lacking basic literacy and cancer mortality (Figure 3 and Figure 4). Basic literacy is also strongly associated with poor outcomes in terms of higher colorectal and lung cancer incidence and mortality. A higher percentage of lacking basic literacy was concentrated in Appalachia, where poor outcomes in terms of increased cancer incidence and mortality exist.

### 3.5. Simple Linear Regression Analysis

We fit a series of simple linear regression models comparing age-adjusted cancer incidence by “did not complete high school” and “lacking basic literacy variables.” We found the association between not completing high school and age-adjusted colorectal and lung cancer incidence was statistically significant (*p* < 0.001) across all pairings (Table 2). Of note, a one percentage point increase in people that did not complete high school correlated significantly with an estimated 1.69 additional cases of lung cancer per 100,000 people (*p* < 0.001). Similarly, a one percentage point increase in lacking basic literacy correlated significantly with an estimated 3.05 additional cases of lung cancer cases per 100,000 people (*p* < 0.001) (Table 2).

### 3.6. Spatial Regression Analysis

We calculated Moran’s I statistic on the residuals of the above simple linear regression models to check for spatial dependence. For did not complete high school, significant spatial autocorrelation was observed for both colorectal (*p* < 0.01) and lung cancer (*p* < 0.05) incidence, but not for mortality of either cancer. For lacking basic literacy, significant spatial autocorrelation was observed for colorectal (*p* < 0.01) and lung cancer (*p* < 0.01) incidence and for lung cancer mortality (*p* < 0.01), but not colorectal cancer mortality. Lagrange multiplier diagnostic tests were used to assess the appropriate spatial model to account for spatial dependence. In all cases, a spatial lag model was indicated and fit (Table 3). Spatial lag estimates account for how rates in a specific county are affected by the independent education variable (direct effect, estimated by Coef in Table 3) and by the independent education variable in neighboring counties (indirect effect). Average total effect can be estimated using the formula Coef/(1-Spatial Parameter) [29]. This tells us, for instance, that the average direct effect of a one percentage point increase in lacking basic literacy for a county was 1.07 more cases of colorectal cancer per 100,000 people (*p* < 0.001). Accounting for spatial dependence, the average total effect of the same one percentage point increase in lacking basic literacy for a county was associated with 1.55 additional cases of colorectal cancer per 100,000 people. We can see that accounting for spatial lag in this case detects a greater impact of lacking basic literacy on colorectal cancer incidence than is detected by simple linear regression alone.

## 4. Discussion

The goal of this study was to examine the relationship between education and colorectal and lung cancer incidence and mortality in non-Appalachian and Appalachian populations. This is the first study to examine the association between cancer outcomes and educational attainment and literacy between Appalachian and non-Appalachian populations at the county level in Kentucky. The results demonstrate significantly elevated cancer incidence and mortality in Appalachian Kentucky populations. The greatest disparity between Appalachian and non-Appalachian populations exists in lung cancer incidence and mortality. The data also shows that low education, described in terms of lack of high school graduation and basic literacy levels, is strongly correlated with high cancer incidence and mortality in Appalachian Kentucky.

Appalachian Kentucky comprises mostly small rural towns. The majority of Kentucky consists of rural counties with a few high-density city centers. Decreased cancer education resources, which are lacking in rural Appalachian regions, may lead to a lack of understanding of cancer preventative measures and an unwillingness to participate in screenings and treatment services [30]. These factors contribute to the higher percentage of preventable cancer malignancies and mortalities in Appalachian Kentucky demonstrated in this study.

### 4.1. Existing Research

Other studies have produced analogous results in different populations. An NCI study examining U.S. adults aged 25–64 used racial and education information to examine how socio-demographic factors such as location and education level factors influence overall cancer-related mortality. The results show that increases in educational attainment are associated with lower overall cancer mortality. Additionally, they found that patients’ educational attainment had a significantly greater impact on cancer risk than racial group identification on all cancer sites except for breast cancer [12]. These findings by Albano and colleagues are corroborated by our findings in that the correlation of educational attainment and age-adjusted overall cancer mortality was similar to that of colorectal and lung cancer.

Furthermore, a study examining age-adjusted cancer-specific incidence with educational attainment in U.S. adults found that adults with lower educational attainment had significantly increased incidence of smoking-related cancers. This demonstrates an inverse relationship between lung cancer mortality and educational attainment, which is consistent with the relationship discovered and presented here [31]. Interestingly, this relationship was more pronounced in states with higher smoking rates, such as Kentucky, Mississippi, and West Virginia [31]. These findings are relevant to those presented here due to the high percentage of smokers concentrated in Appalachian Kentucky. Recent reports state that Kentucky ranks second in the nation for smoking prevalence among adult populations, [32] which greatly contributes to the higher lung cancer rates. States with higher smoking rates see significantly increased lung cancer-related mortality, and this disparity is further exacerbated by the low educational attainment in these rural states [33]. Studies conducted in China found that individuals that graduated from college or above had a 58% reduction in the odds of being diagnosed with colorectal cancer (OR 0.42, 95% CI: 0.34–0.52) compared to individuals that completed primary school or below, which agrees with our findings [34]. Additionally, a cross-sectional study in California found that participants with only a high school education had between 8 and 45% higher CRC mortality than their counterparts achieving above a high school education [35].

However, our study is the first to evaluate the relationship between education and colorectal cancer in a largely rural population and comparing Appalachian and non-Appalachian populations. Furthermore, our bivariate spatial visualizations of county-level data elucidated new insights into the geographic distribution of co-occurring low education with high incidence or mortality for lung and colorectal cancers, which showed a notably higher presence of these counties in Appalachia. Our findings also highlighted the importance of further investigating the ways in which educational attainment and literacy may influence cancer incidence and mortality outcomes via their impact on cancer screening and health risk behaviors.

### 4.2. Application of Findings

We found that as educational attainment decreases, cancer incidence and mortality rates increase. Research has shown that 49% of those lacking a high school diploma show restricted health literacy [18]. Increased health literacy levels in patients with bachelor’s degrees can also lead to greater participation in health behaviors linked to cancer prevention, screening, and treatment [18,36]. Additionally, adults living below the poverty line have significantly reduced cancer literacy when compared to those living at or above the poverty line [18]. Poverty levels in Kentucky are significantly elevated when compared to the remainder of the U.S. [37]. Because educational attainment in Appalachian Kentucky is low and poverty levels are high, these citizens may be more likely to lack an understanding of the importance of preventive measures, which contributes to the increased colorectal and lung cancer incidence and mortality rates. Targeting efforts at reducing high school dropout rates, increasing college enrollment and completion, and improving health literacy, especially cancer literacy, among school-aged youth and young adults could help to reduce the burden of colorectal and lung cancer in Appalachia over time. The bivariate spatial visualizations can be used to target resources in the counties with the highest risk.

Future research could use individual-level data to attempt to untangle the direct and indirect effects of education on colorectal cancer and lung cancer via the intermediate effects on cancer screening, smoking, and other individual behaviors. In addition, future study directions should explore how this relationship between education and cancer incidence and mortality can be used to inform cancer-related interventions. Studies have demonstrated that a brief cancer-related intervention can significantly increase short and long-term cancer knowledge in Appalachian Kentucky youth [38,39] and a cancer education curriculum has been created for this population [40]. Additional research is necessary to explore incorporating a cancer education curriculum into Appalachian schools and research is needed to determine how legislative actions in terms of policies and/or the funding of cancer prevention and control interventions may improve cancer literacy and reduce incidence and mortality in Appalachia.

### 4.3. Strengths and Limitations

This is the first study to explore the relationship between colorectal and lung cancer mortality and incidence rates and education measures in Appalachian and non-Appalachian counties in Kentucky. A strength of this study is the direct comparability of education attainment due to state Department of Education standards that apply to all Kentucky schools, including high school graduation. While this study shows that education is an important social determinant of cancer outcomes, there are some limitations. First, we present SEER data from Kentucky residents based on county-level data using ecological analysis, which shows informative patterns across geographic levels, but cannot be directly inferred to individual behavior or health outcomes, as that could lead to an ecological fallacy [41]. Second, due to the unique context of each state, these findings may not be directly generalizable to Appalachian counties in other states. Third, while we report education as a social determinant of health and cancer risk, other social determinants of health such as income, poverty, geography, and access to opportunities may also have varying influence on cancer incidence and mortality. Finally, in utilizing SEER data, there is a systematic error in data quality based on the variation of data collection due to geographic location, and SEER cancer mortality data may have missing, incomplete, or inaccurate causes of death leading to the underestimation of cancer mortality.

## 5. Conclusions

The highest and most substantial disparities in cancer incidence and mortality rates exist in Kentucky and are concentrated in the Appalachian region of Kentucky. Furthermore, the Appalachian region of Kentucky lags behind the nation and non-Appalachian regions in educational attainment. Low educational attainment coupled with low health literacy likely further exacerbates the burden of cancer in Appalachian Kentucky. Our finding that low educational attainment was strongly correlated with high cancer incidence in Appalachian Kentucky supports this. Furthermore, we show that lacking basic literacy is strongly correlated with higher cancer incidence and mortality. Implementing interventions to increase educational attainment may enhance health literacy and low-literacy health education interventions could contribute to reducing cancer incidence and mortality rates in Appalachia, diminishing disparities in the region. Funding initiatives that support increasing educational attainment and implementing policies that implement cancer education into middle and high school curriculums in Kentucky, more specifically Appalachian communities, may also contribute to reducing the burden of colorectal and lung cancer.

## Figures and Tables

**Table 1:** Age-adjusted colorectal and lung cancer incidence and mortality (per 100,000 people) in non-Appalachian Kentucky vs. Appalachian Kentucky populations, 2014–2018.

Measure	Site	Appl KY Rate	Non-Appl KY Rate	Rate Ratio (Ref. Non-Appl KY)	% Higher in Appl KY
Incidence	All Site	538.6	510.0	1.06 ***	5.60
	Colorectal	54.8	45.4	1.21 ***	20.70
	Lung	105.3	82.4	1.28 ***	27.79
Mortality	All Site	172.3	153.8	1.12 ***	12.02
	Colorectal	15.3	13.5	1.13 ***	13.33
	Lung	48.4	37.5	1.29 ***	29.07

Data sources are from reference [28]. A rate ratio approach was used to test for two-sided statistical difference between two age-adjusted rates. *** *p* < 0.001.

**Table 2:** Simple linear regression analysis expressing linear coefficient of a linear model comparing age-adjusted colorectal or lung cancer incidence or mortality (per 100,000 people) by did not complete high school or basic literacy.

Age-Adjusted Cancer Rates per 100,000 People	Cancer Site	Did Not Complete High School			Lacking Basic Literacy		
		Coef	Std Coef	R²	Coef	Std Coef	R²
Incidence	Colorectal	0.62 ***	0.37 ***	13.9%	1.35 ***	0.39 ***	14.9%
	Lung	1.69 ***	0.61 ***	37.6%	3.05 ***	0.53 ***	27.7%
Mortality	Colorectal	0.30 ***	0.37 ***	14.0%	0.58 ***	0.34 ***	11.8%
	Lung	1.45 ***	0.64 ***	40.8%	2.78 ***	0.58 ***	34.1%

* *p* < 0.05; ** *p* < 0.01; *** *p* < 0.001. Coef = Unstandardized coefficient; Std Coef = Standardized coefficient.

**Table 3:** Spatial regression analysis expressing coefficient of spatial lag models comparing age-adjusted colorectal or lung cancer incidence or mortality (per 100,000 people) by did not complete high school or basic literacy.

Age-Adjusted Cancer Rates per 100,000 People	Cancer Site	Did Not Complete High School			Lacking Basic Literacy		
		Coef	Std Coef	Spatial Parameter	Coef	Std Coef	Spatial Parameter
Incidence	Colorectal	0.47 ***	0.29 ***	0.30 *	1.07 ***	0.31 ***	0.31 **
	Lung	1.36 ***	0.50 ***	0.28 *	2.23 ***	0.38 ***	0.40 ***
Mortality	Colorectal	0.27 ***	0.34 ***	0.16	0.51 ***	0.30 ***	0.20
	Lung	1.16 ***	0.51 ***	0.28 *	2.09 ***	0.44 ***	0.38 ***

* *p* < 0.05; ** *p* < 0.01; *** *p* < 0.001. Coef = Unstandardized coefficient; Std Coef = Standardized coefficient.

## Data Availability

The datasets generated during and/or analyzed during the current study are publicly available as described in the methods section. In short, the cancer incidence and mortality data were obtained from the Kentucky Cancer Registry (KCR), a population-based central cancer registry for the Commonwealth of Kentucky and member of the National Cancer Institute’s Surveillance, Epidemiology, and End Results (SEER) program since 2000 (accessed from https://www.cancer-rates.info/ky/ on 29 June 2021). County level population percentages of those age 25 or older who did not attend high school, who did not complete high school, and who did not complete college were gathered from the American Community Survey 5-Year Estimates for 2014–2018 (accessed from https://data.census.gov/cedsci/ on 12 June 2023). Basic literacy were obtained from the 2003 National Assessment of Adult Literacy (NAAL) (accessed from https://nces.ed.gov/naal/ on 18 July 2021).
